# Cytokine Response of Cultured Skeletal Muscle Cells Stimulated with Proinflammatory Factors Depends on Differentiation Stage

**DOI:** 10.1155/2013/617170

**Published:** 2013-02-20

**Authors:** Matej Podbregar, Mitja Lainscak, Oja Prelovsek, Tomaz Mars

**Affiliations:** ^1^Centre for Intensive Care Medicine, University Medical Centre Ljubljana, 1000 Ljubljana, Slovenia; ^2^Division of Cardiology, University Clinic of Respiratory and Allergic Diseases Golnik, 4204 Golnik, Slovenia; ^3^Applied Cachexia Research, Department of Cardiology, Charité Medical School, Campus Virchow-Klinikum, Berlin, Germany; ^4^Institute of Pathophysiology, Faculty of Medicine, University of Ljubljana, Zaloska Cesta 4, 1000 Ljubljana, Slovenia

## Abstract

Myoblast proliferation and myotube formation are critical early events in skeletal muscle regeneration. The attending inflammation and cytokine signaling are involved in regulation of skeletal muscle cell proliferation and differentiation. Secretion of muscle-derived cytokines upon exposure to inflammatory factors may depend on the differentiation stage of regenerating muscle cells. Cultured human myoblasts and myotubes were exposed to 24-hour treatment with tumor necrosis factor (TNF)-**α** or lipopolysaccharide (LPS). Secretion of interleukin 6 (IL-6), a major muscle-derived cytokine, and interleukin 1 (IL-1), an important regulator of inflammatory response, was measured 24 hours after termination of TNF-**α** or LPS treatment. Myoblasts pretreated with TNF-**α** or LPS displayed robustly increased IL-6 secretion during the 24-hour period after removal of treatments, while IL-1 secretion remained unaltered. IL-6 secretion was also increased in myotubes, but the response was less pronounced compared with myoblasts. In contrast to myoblasts, IL-1 secretion was markedly stimulated in LPS-pretreated myotubes. We demonstrate that preceding exposure to inflammatory factors stimulates a prolonged upregulation of muscle-derived IL-6 and/or IL-1 in cultured skeletal muscle cells. Our findings also indicate that cytokine response to inflammatory factors in regenerating skeletal muscle partially depends on the differentiation stage of myogenic cells.

## 1. Introduction

Skeletal muscle normally represents 40% of body weight and has a vital role in locomotion and whole body metabolism. Disorders associated with loss of skeletal muscle, including cancer cachexia and age-related sarcopenia, are associated with increased morbidity and mortality [1–4]. Muscle regeneration is vital for maintenance of skeletal muscle mass and function [5]. Different stimuli, including overloading, denervation, direct injury to muscle fibers, or different clinical conditions, trigger regeneration process by activating muscle satellite cells, which transform into proliferating myoblasts. Subsequently, myoblasts fuse with existing muscle fibers or fuse with each other to form multinucleated myotubes [5, 6]. As well as reconstituting muscle tissue following major injury, regeneration process constantly removes smaller lesions due to daily wear-and-tear [7]. Furthermore, satellite cell activation and myoblast proliferation are involved in load-induced muscle hypertrophy [8]. Indeed, myoblasts may contribute new nuclei to existing muscle fibers and thereby support the hypertrophic muscle growth [8, 9]. Additionally other stem cell populations have been identified recently and can participate in muscle regeneration and growth [10]. This indicates that exercise-induced or pharmacological stimulation of muscle regeneration could represent a strategy to treat muscle atrophy associated with different diseases. 

Skeletal muscle is an important source of interleukin-6 (IL-6) and other muscle-derived cytokines (myokines), which modulate immune responses and regulate energy metabolism [11]. In skeletal muscle IL-6 is lowly expressed under resting conditions, but is markedly induced during exercise [11, 12]. IL-6 expression is also up-regulated in regenerating skeletal muscle [13, 14]. Moreover, IL-6 is constitutively secreted in myogenic precursor cells, including myoblasts and myotubes [15, 16]. IL-6 promotes myoblast proliferation [8, 17] and/or myotube formation [18, 19], suggesting a role for IL-6 in muscle regeneration. Additionally, by stimulating myoblast proliferation IL-6 may contribute to load-induced muscle hypertrophy [8]. Regulation of IL-6 secretion during muscle regeneration has not been fully characterized, but may involve inflammatory cytokines, including tumor necrosis factor *α* (TNF-*α*) and interleukin-1 (IL-1). 

Although chronic exposure to TNF-*α* and IL-1 leads to insulin resistance and proteolysis in skeletal muscle [20–22], both salient characteristics of cachectic states [2], TNF-*α* and IL-1 could directly or indirectly promote early stages of skeletal muscle regeneration. First of all, TNF-*α* and IL-1 as well as their receptors are up-regulated in regenerating skeletal muscle [23]. Furthermore, during the initial stages of muscle regeneration macrophages accumulate in injured muscle and represent an important source of TNF-*α* and IL-1 [5, 24]. As well as removing necrotic debris, macrophages are thought to regulate muscle regeneration directly by stimulating proliferation and/or differentiation of myogenic precursor cells [5, 25]. Also, TNF-*α* and IL-1 are well-characterized upstream regulators of IL-6 secretion under septic conditions [26, 27] and in cultured skeletal muscle cells [15, 28], suggesting they may indirectly stimulate muscle regeneration by enhancing IL-6 mediated signaling. Finally, TNF-*α* was shown to promote and/or sustain proliferation [29, 30]. However, the understanding of the role of IL-1 and TNF-*α* in orchestrating muscle regeneration is incomplete. 

During regeneration skeletal muscle cells proceed through an extensive developmental program, which transforms proliferating mononuclear myoblasts into terminally differentiated multinucleated muscle fibers [6]. Considering fundamental phenotypic differences between myoblasts and mature muscle fibers, responsiveness to inflammatory cytokines may depend on the differentiation stage of skeletal muscle cells. The aim of this study was to determine whether endogenous or exogenous inflammatory factors differentially regulate secretion of muscle-derived cytokines in different developmental stages of cultured skeletal muscle. 

## 2. Materials and Methods

### 2.1. Reagents

ELISA kits for human IL-6 and IL-1 were from Pierce/Thermo Scientific (Waltham, MA, USA). Cell culture flasks and plates were obtained from BD Falcon (Franklin Lakes, NJ, USA). Advanced Minimal Essential Medium (MEM), fetal bovine serum (FBS), Earle's Balanced Salt Solution, trypsin, gentamycin, and Fungizone were obtained from Invitrogen (Paisley, UK). Hoechst was from Molecular Probes (Invitrogen) and Cytotoxicity Detection kit (LDH) from Roche Applied Science (Mannheim, Germany). All other reagents, including lipopolysaccharide (LPS) and TNF-*α*, were of analytical grade and were purchased from Sigma-Aldrich unless otherwise specified. 

### 2.2. Human Skeletal Muscle Cell Culture

This study was approved by the Ethical Commission at the Ministry of Health of the Republic of Slovenia (no. 63/01/99) and (no. 71/05/12). Human skeletal muscle cultures were prepared from muscle tissue samples obtained during routine orthopaedic surgery from donors without neuromuscular disease. All donors gave their written informed consent. Preparation of primary culture of human skeletal muscle cells was performed as previously described [31–34]. Briefly, muscle samples were cleaned of connective and adipose tissue, cut into small pieces, and then trypsinised for 30 minutes in Earle's Balanced Salt Solution supplemented with trypsin-EDTA at 37°C. The released skeletal muscle cells were grown on Petri dishes in growth medium (Advanced MEM supplemented with 10% (v/v) FBS, 0.3% (v/v) Fungizone and 0.15% (v/v) gentamycin) at 37°C in 5% CO_2_/humidified air. After 2-3 weeks and before fusion into myotubes myoblast colonies were selectively trypsinised and transferred to 75 cm^2^ cell culture flasks. Growth medium was changed 2-3 times per week and myoblasts were always subcultured before reaching confluence. They were propagated for 2-3 passages before being used for experiments. All experiments were performed on skeletal muscle cell cultures from 4–6 donors.

### 2.3. TNF-*α* and LPS Treatments in Cultured Myoblasts and Myotubes

Before the experiment, myoblasts were seeded in six-well plates (BD Falcon), where they were grown on glass cover slips and coated with a 1 : 2 mixture of 1.5% gelatin (Sigma, St. Louis, MI, USA) and human plasma. Experiments on myoblasts were performed on subconfluent cultures before the start of fusion into myotubes. To induce myogenic differentiation and fusion into myotubes, subconfluent myoblast cultures were switched from growth medium to differentiation medium (Advanced MEM supplemented with 2% FBS, 0.3% (v/v) Fungizone and 0.15% (v/v) gentamycin) for 2-3 days. When myotubes started to form, differentiation medium was exchanged for growth medium. Experiments on myotubes were carried out after 3 weeks of differentiation. Cultured myoblasts or myotubes were treated with 100 ng/mL TNF-*α* (Sigma) or 100 ng/mL LPS (Sigma) or vehicle in growth medium for 24 hours. On the second day of experiment media containing treatments were replaced with growth medium (washout). Experiment was terminated 24 hours later, when media were collected for determination of secreted cytokines.

### 2.4. Determination of IL-6 and IL-1 Secretion from Cultured Human Myoblasts and Myotubes

Concentrations of IL-6 and IL-1 in cell culture media were measured with human IL-6 and IL-1 ELISA kits (Pierce/Endogen Thermo Scientific) according to the manufacturer's instructions. Growth medium was used as a diluent for the standards as well as the samples to avoid analytical interference. Data were normalized to the number of nuclei per well to allow comparison of cytokine secretion between mononuclear myoblasts and polynuclear myotubes, as described [15]. Briefly, cell cultures on cover slips were fixed with 4% paraformaldehyde in phosphate-buffered saline (pH 7.4) for 15 min. They were subsequently exposed to 1 mM Hoechst 33258 (Molecular Probes-Life Technologies, Willow creek, OR, USA), prepared in phosphate-buffered saline, for 5 min. Number of nuclei per well was estimated by counting Hoechst-stained nuclei in 10 random fields of view per well at 200x magnification. Cytokine secretion data are expressed as the mass of secreted IL-1 or IL-6 in ng/24 h/100,000 nuclei. 

### 2.5. Assessment of Cytotoxicity and Apoptosis

Cytotoxicity was assessed by measuring the activity of lactate dehydrogenase (LDH) in cell culture medium using Cytotoxicity Detection kit (LDH) (Roche Applied Science, Mannheim, Germany) according to manufacturer's instructions. TNF-*α* and LPS cause apoptosis in some cell types we tested our cultures for this effect. Apoptosis was assessed by the DNA fragmentation and nuclear chromatin examination, as described [15]. For DNA fragmentation we harvest cells treated with TNF-*α* and LPS; total DNA was isolated using the Wizard Genomic DNA Purification Kit (Promega, Madison, WI, USA). Electrophoresis was performed on ethidium-bromide stained 1.8% agarose gel and visualized with transilluminator. To obtain a positive control of the DNA ladder, cells were treated with 10% DMSO for three days. For nuclear chromatin examinations, cells, grown on sterile collagen-coated coverslips, or eventually floating dead cells were fixed in phosphate-buffered saline (PBS) containing 4% paraformaldehyde, stained with 1 mM Hoechst 33258 in PBS and examined under fluorescence microscope. Cells were scored as apoptotic if they exhibited unequivocal nuclear chromatin condensation and/or fragmentation.

### 2.6. Statistics

The data are presented as means ± SEM. Univariate two-way or three-way analysis of variance (ANOVA) was used to analyze the differences between the myoblasts and myotubes for their IL-6 and IL-1 secretion under the different experimental conditions. Bonferroni post hoc test was used. Statistical significance was established at *P* < 0.05. The data were analyzed using SPSS 13.0 for Windows (SPSS Inc., Chicago, IL, USA) and Microsoft Excel (Microsoft Office Excel 2003). 

## 3. Results

### 3.1. IL-6 Secretion in Cultured Myoblasts and Myotubes Is Increased after Removal of TNF-*α* and LPS

**Figure 1:**
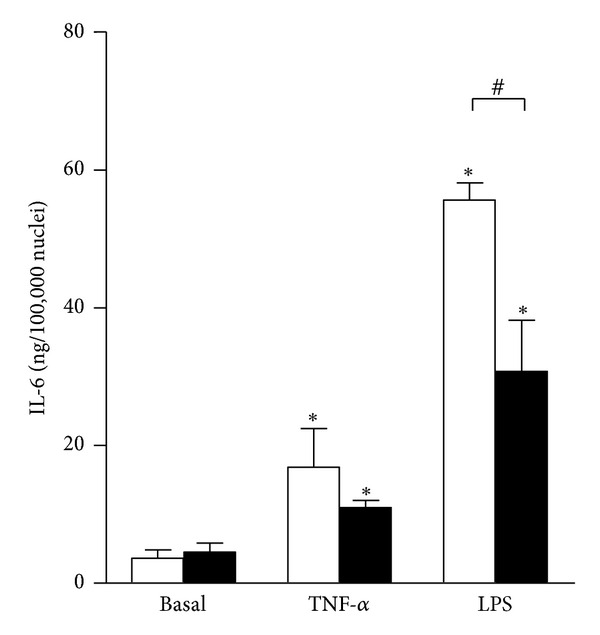
IL-6 secretion from cultured skeletal muscle cells is increased after removal of TNF-*α* and LPS treatment. Human myoblasts (white bars) and myotubes (black bars) were exposed to TNF-*α* (100 ng/mL) or LPS (100 ng/mL) or vehicle (Basal) for 24 hours. Medium containing TNF-*α* or LPS was removed and replaced with growth medium. IL-6 secretion was estimated by ELISA 24 hours after compound removal. Data are means ± SEM (*n* = 4–6). *P < 0.05 versus respective Basal. ^#^ *P* < 0.05 myoblasts versus myotubes (95% confidence interval (CI) of difference: −39.96 to −9.74).

Initial stages of skeletal muscle regeneration are characterized by the presence of proinflammatory macrophages, which secrete TNF-*α* and IL-1 [5]. Later macrophages downregulate TNF-*α* and IL-1 expression and acquire anti-inflammatory properties, which is thought to underlie resolution of the inflammation [5, 25]. We have previously demonstrated that myoblast and myotubes increase IL-6 secretion in response to treatment with TNF-*α* [15]. To explore whether preceding exposure to TNF-*α* affects cytokine secretion in cultured skeletal muscle cells, myoblasts and myotubes were treated with 100 ng/mL TNF-*α* or vehicle for 24 hours. Treatment media were subsequently removed and fresh growth medium was added. Cytokine secretion was assessed during the 24 hours after removal of TNF-*α* (Figure 1). Basal IL-6 secretion was similar between myoblasts and myotubes. Increased IL-6 secretion was detected in myoblasts (*P* < 0.05) and myotubes (*P* < 0.05) pretreated with TNF-*α*. Secretion of IL-6 tended to be lower in myotubes, but the difference did not reach the level of statistical significance. Under *in vitro *conditions, macrophages can be induced to acquire pro-inflammatory phenotype by bacterial lipopolysaccharide (LPS) [25]. LPS also directly stimulates IL-6 secretion from cultured myoblasts and myotubes [15]. We therefore determined whether 24-hour pretreatment with 100 ng/mL LPS affects IL-6 secretion in cultured myoblasts and myotubes (Figure 1). Pretreatment with LPS resulted in a robustly increased IL-6 secretion from myoblasts (*P* < 0.05) and myotubes (*P* < 0.05) during 24 hours after removal of LPS. Consistent with TNF-*α* treatment, myoblasts were more responsive to stimulation with LPS compared to myotubes (*P* < 0.05). These results indicate that preceding treatment with TNF-*α* and LPS has a prolonged effect on IL-6 secretion in cultured skeletal muscle.

### 3.2. IL-1 Secretion in Cultured Myoblasts and Myotubes Is Increased after Removal of TNF-*α* and LPS

**Figure 2:**
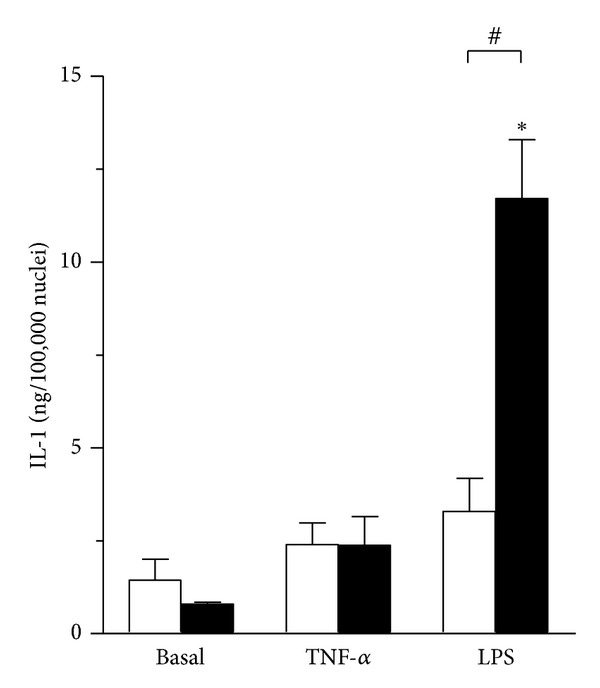
IL-1 secretion from cultured skeletal muscle cells is increased after removal of TNF-*α* and LPS treatment. Human myoblasts (white bars) and myotubes (black bars) were exposed to TNF-*α* (100 ng/mL) or LPS (100 ng/mL) or vehicle (Basal) for 24 hours. Medium containing TNF-*α* or LPS was removed and replaced with growth medium. IL-1 secretion was estimated by ELISA 24 hours after compound removal. Data are means ± SEM (*n* = 4–6). *P < 0.05 versus respective Basal. ^#^ *P* < 0.05 myoblasts versus myotubes (95% CI of difference: 5.28 to 11.56).

Next we investigated whether TNF-*α* and LPS affect secretion of muscle-derived IL-1 in cultured myoblasts and myotubes. IL-1 has a well-characterized role in LPS-triggered cytokine cascade under septic conditions [27], but IL-1 is usually not recognized as a member of myokine family and its function during skeletal muscle regeneration is poorly understood. Cultured myoblasts and myotubes robustly secreted IL-1 in amounts comparable to IL-6 (Figure 2). Pre-treatment with TNF-*α* tended to increase secretion of IL-1 from myoblasts and myotubes, but the increase did not reach the level of statistical significance (Figure 2). In sharp contrast, LPS pre-treated myotubes displayed a robust increase in IL-1 secretion (*P* < 0.05) during 24 hours after removal of LPS. Myotubes were markedly more responsive to LPS pretreatment compared with myoblasts (*P* < 0.05). These data suggest IL-1 secretion in regenerating myoblasts is not increased upon removal of pro-inflammatory factors.

### 3.3. Cytokine Response Upon Exposure to Proinflammatory Factors Depends on Differentiation Stage of Cultured Skeletal Muscle Cells

**Figure 3:**
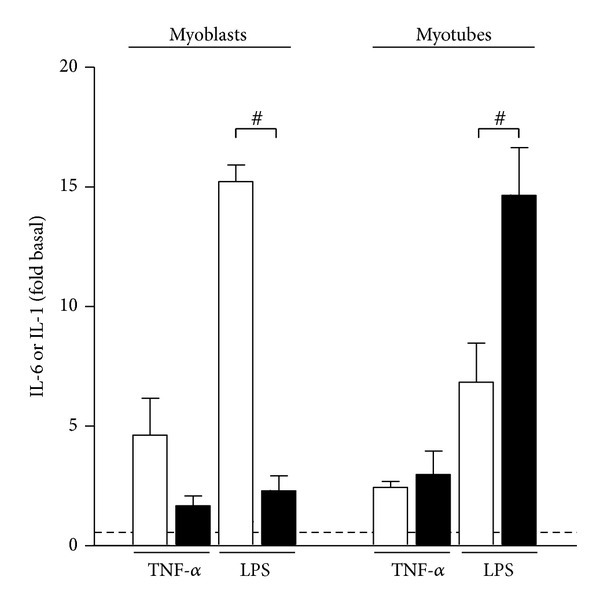
Cultured skeletal muscle cells are characterized by differential responsiveness to pro-inflammatory factors. Human myoblasts and myotubes were exposed to TNF-*α* (100 ng/mL) or LPS (100 ng/mL) for 24 hours. Medium containing TNF-*α* or LPS was removed and replaced with growth medium. Fold increase in IL-6 (white bars) and IL-1 (black bars) secretion following exposure to TNF-*α* or LPS was calculated to assess responsiveness of myoblasts and myotubes to stimulation with pro-inflammatory factors TNF-*α* or LPS. The dashed line symbolically represents the level of IL-6 or IL-1 secretion under basal conditions. Data are means  ± SEM (*n* = 4–6). ^#^ *P* < 0.05 versus IL-6 secretion in LPS-treated myoblasts (95% CI of difference: −18.34 to −7.53) and myotubes (95% CI of difference: 2.42 to 13.23).

Proliferating myoblasts and differentiated myotubes are two distinct developmental stages, characterized by fundamental phenotypic differences. IL-6 promotes myoblast proliferation [8, 17], whereas IL-1 has a prominent role in immune response and may act as a negative regulator of myogenic differentiation [35]. To assess the potential differences in cytokine response in myoblasts and myotubes display different, we analyzed IL-1 and IL-6 secretion pattern following exposure to TNF-*α* and LPS (Figure 3). Myoblasts and myotubes were responsive to both TNF-*α* and LPS, but their response pattern was markedly different. TNF-*α* and LPS pretreatment in myoblasts resulted in robustly increased IL-6 secretion, whereas IL-1 was not appreciably increased. By contrast, in LPS-treated myotubes IL-1 was more strongly induced than IL-6 (*P* < 0.05). IL-6 and IL-1 secretion in myotubes was higher following LPS pretreatment compared with TNF-*α* (*P* < 0.05). Taken together our data suggest that pro-inflammatory factors induce prolonged up-regulation of IL-6 in myoblasts, whereas myotubes display a robust increase in IL-1 secretion concomitant with less pronounced stimulation IL-6 production.

### 3.4. TNF-*α* and LPS Treatment Did Not Induce Cell Death

LPS and TNF-*α* treatment can lead to cytotoxicity and cell death [36]. Cytotoxicity of TNF-*α* and LPS treatment was assessed by measuring the activity of lactate dehydrogenase in cell culture media as describes in Materials and Methods. There was no difference in lactate dehydrogenase activity in myoblasts or myotubes under basal condition compared to TNF-*α* and LPS treatment (data not shown). Apoptosis was not induced, as assessed by evaluation of DNA fragmentation and chromatin condensation. Also, the number of cells per well was similar between different treatments.

## 4. Discussion

Skeletal muscle regeneration is an essential process for maintenance of muscle mass and function [5, 6]. Although exposure to pro-inflammatory cytokines like TNF-*α* and IL-1 may has deleterious effects on skeletal muscle [2, 20, 21], cytokine signaling plays an important role in skeletal muscle regeneration and/or hypertrophy [5, 6, 8, 25]. TNF-*α* and IL-1 are produced by immature skeletal muscle cells [15, 16, 37] and/or by macrophages, which infiltrate injured skeletal muscle [5, 25]. Moreover, evidence suggests TNF-*α* stimulates and/or prolongs myoblast proliferation [29, 30], indicating that TNF-*α* may promote muscle regeneration. Molecular mechanisms underlying divergent roles of pro-inflammatory cytokines in skeletal muscle remain to be established, but differential responsiveness to external stimuli could depend on the developmental stage of skeletal muscle cells during regeneration and on dosage and time of exposure. Here we show that proliferating myoblasts respond to pro-inflammatory factors TNF-*α* and LPS with prolonged increase in IL-6 secretion, while IL-1 secretion remained unaltered. By contrast myotubes displayed markedly increased IL-1 secretion, but exhibited less pronounced IL-6 up-regulation.

Skeletal muscle is an important source of cytokines, which regulate many aspects of muscle function and participate in regulation of immune responses as well as whole body metabolism [11, 38]. We observed robust IL-6 secretion under basal conditions, which is consistent with earlier studies in cultured skeletal muscle cells [15, 16, 39]. Several factors are known to modulate IL-6 secretion from skeletal muscle cells, including LPS, TNF-*α*, glucocorticoids, and hypoxia [15, 16, 33], but regulatory mechanisms underlying basal IL-6 secretion are incompletely understood. Interestingly, myoblasts and myotubes constitutively secreted IL-1 in amounts comparable to IL-6, whereas substantially lower level of IL-1 secretion was previously observed in cultured myoblasts [16]. However, we did not detect increased IL-1 production in myoblasts exposed to TNF-*α* or LPS although IL-6 secretion was markedly stimulated. This suggests that putative autocrine stimulation by IL-1 does not play a major role in inflammatory factor-induced up-regulation of IL-6 in cultured skeletal muscle cells. Of note, endogenous TNF-*α* expression was observed in C2C12 cells under basal conditions [37]. Similarly, we detected modest secretion of TNF-*α* in cultured human skeletal muscle cells (data not shown). Taken together this indicates that endogenous TNF-*α* production could represent an additional stimulus for basal IL-6 secretion in myoblasts and myotubes [15].

Early stages of muscle regeneration are characterized by increased expression of TNF-*α* and IL-1 as well as LPS-binding protein [23]. This is coincident with influx of neutrophils and macrophages into the injured skeletal muscle. Initially, resident macrophages display inflammatory phenotype and secrete pro-inflammatory cytokines like TNF-*α* and IL-1 [5]. Subsequently, macrophages suppress TNF-*α* and IL-1 expression and acquire an anti-inflammatory phenotype, which is thought to be particularly important for the outcome of regeneration process [5, 25]. We previously demonstrated that pro-inflammatory milieu, mimicked by TNF-*α* or LPS treatment strongly induces IL-6 secretion in cultured myoblasts and myotubes [15]. In the work presented here we determined whether preceding exposure to pro-inflammatory environment has a prolonged effect on IL-6 up-regulation. Indeed, stimulation of cultured myoblasts by TNF-*α* or LPS led to prolonged increase in IL-6 secretion even as pro-inflammatory treatment was removed. We also found that cultured myotubes robustly increase IL-1 production in response to LPS, whereas IL-1 secretion remained unaltered in myoblasts. As previously reported, TNF-*α* exposure did not increase IL-1 secretion from cultured myoblasts [16]. These data demonstrate that cytokine response in cultured human skeletal muscle cells persists even after removal of pro-inflammatory factors, indicating withdrawal of inflammatory stimuli may not be sufficient for immediate resolution of inflammation associated with skeletal muscle injury and regeneration. These findings are also compatible with the notion that switches to pro-inflammatory macrophage phenotype, which involves secretion of anti-inflammatory cytokines, actively promotes termination of acute inflammation [5, 25].

Our data show that, in contrast to constitutive level of cytokine secretion, the production of cytokines following exposure to inflammatory factors TNF-*α* and LPS is related to the developmental stage of myogenic cells. This is in agreement with our previous observations that induction of IL-6 secretion in human myoblasts during TNF-*α* or LPS treatment is more pronounced compared with response in myotubes [15]. The biological basis of these differences is not fully understood but could reflect phenotypic differences between myoblasts and myotubes. Mononuclear myoblasts are actively proliferating cells, which markedly differ from the differentiated multinucleated myotubes as regards protein expression and responses to various extrinsic factors [40]. Moreover, while myoblasts proliferate intensively in order to reach the critical number of cells that are necessary for successful fusion, myotubes are destined for further differentiation, innervation and maturation to adult muscle fibers [5, 6, 41]. The distinctive biological difference in the role played by myoblasts and myotubes during muscle regeneration is reflected in different expression patterns of TNF-*α* receptors [42] and Toll-like receptors [43] in myoblasts and myotubes, with consecutive activation of different intracellular signaling pathways during muscle ontogenesis and regeneration [7, 44]. 

Inflammation during initial stages of regeneration has an important role in restoration of muscle function after injury [5, 24]. However, chronic inflammation and/or exposure to pro-inflammatory cytokines has deleterious effect on skeletal muscle [2, 20, 21, 45]. Timely suppression of inflammation by anti-inflammatory macrophages is therefore considered crucial for a favorable outcome of regeneration [5, 25]. Here we demonstrate increased IL-6 and IL-1 secretion even after removal of TNF-*α* and LPS treatment. Considering that IL-6 increases myoblast proliferation [8, 17], this indicates pro-inflammatory factors could promote early stages of muscle regeneration by indirectly increasing the number of myogenic cells. Additionally, TNF-*α* may directly increase myoblast proliferation [30]. However some studies demonstrated that TNF-*α* may have also inhibitory effect upon myoblast differentiation [46–48]. In contrast to IL-6, increased IL-1 secretion was detected only in myotubes. IL-1 is an important mediator of the immune response and plays a role in the local response to infection and tissue injury as well as regulation of cell proliferation, differentiation and apoptosis [49], but its role in muscle regeneration is not well-characterized. Thus, increased IL-1 production upon exposure to inflammatory factors may concomitantly reduce myotube formation and promote IL-6-stimulated myoblast proliferation. Transient stimulation with pro-inflammatory factors may therefore promote myogenesis by increasing the number of myoblasts, whereas diminished differentiation of myogenic cells would tend to impair the outcome of regeneration during chronic exposure to pro-inflammatory factors. Altogether, our findings are compatible with the observations that pro-inflammatory (TNF-*α* and IL-1 expressing) macrophages stimulate myoblast proliferation even as they suppress myoblast differentiation and fusion into myotubes [5, 25].

Collectively, our findings demonstrate that skeletal muscle cells display different cytokine response patterns upon stimulation with endogenous or exogenous pro-inflammatory factors TNF-*α* or LPS, respectively. Moreover, we show that TNF-*α* or LPS treatment has a prolonged effect on increased cytokine secretion in cultured myoblasts and myotubes even as pro-inflammatory treatment is terminated. Although myoblasts and myotubes display similar basal IL-1 and IL-6 secretion, myoblasts tend to respond to pro-inflammatory stimuli with increased IL-6 secretion, whereas IL-1 production was particularly strongly augmented in myotubes. Regulation of cytokine production therefore depends on the differentiation stage and is probably cytokine-type specific. These findings shed further light on the complex interactions between the cytokines and skeletal muscle cells during regeneration.
